# Robust long-read native DNA sequencing using the ONT CsgG Nanopore system

**DOI:** 10.12688/wellcomeopenres.11246.3

**Published:** 2018-08-30

**Authors:** Jean-Michel Carter, Shobbir Hussain

**Affiliations:** 1 Department of Biology and Biochemistry, University of Bath, Bath, BA2 7AY, UK

**Keywords:** Nanopore sequencing, long read sequencing, CsgG, R9.4, Oxford Nanopore, complex genomes, HAP1

## Abstract

*Background: *The ability to obtain long read lengths during DNA sequencing has several potentially important practical applications. Especially long read lengths have been reported using the Nanopore sequencing method, currently commercially available from Oxford Nanopore Technologies (ONT). However, early reports have demonstrated only limited levels of combined throughput and sequence accuracy. Recently, ONT released a new CsgG pore sequencing system as well as a 250b/s translocation chemistry with potential for improvements. *Methods:* We made use of such components on ONTs miniature ‘MinION’ device and sequenced native genomic DNA obtained from the near haploid cancer cell line HAP1. Analysis of our data was performed utilising recently described computational tools tailored for nanopore/long-read sequencing outputs, and here we present our key findings. *Results:* From a single sequencing run, we obtained ~240,000 high-quality mapped reads, comprising a total of ~2.3 billion bases. A mean read length of 9.6kb and an N50 of ~17kb was achieved, while sequences mapped to reference with a mean identity of 85%. Notably, we obtained ~68X coverage of the mitochondrial genome and were able to achieve a mean consensus identity of 99.8% for sequenced mtDNA reads. *Conclusions: *With improved sequencing chemistries already released and higher-throughput instruments in the pipeline, this early study suggests that ONT CsgG-based sequencing may be a useful option for potential practical long-read applications with relevance to complex genomes.

## Introduction

In 1977, Sanger and colleagues at the Laboratory for Molecular Biology in Cambridge, England, described an efficient sequencing-by-synthesis (SBS) approach to determine the order of nucleobases in DNA molecules ( Sanger *et al.*, 1977). This event would go on to revolutionise genetics research, including enabling the first near-complete sequencing of the human genome ( Lander *et al.*, 2001; Venter *et al.*, 2001). The practical success of the method is currently perhaps equalled only by another SBS-based technique, also developed in Cambridge by Balasubramanian, Klenerman and colleagues ( Bentley *et al.*, 2008), and which was later taken on to huge commercial success by the biotech company Illumina. While Illumina sequencing, as well as other second generation sequencing methods, enable high-throughput yields with very good to excellent accuracy, they all suffer from the major drawback of short read lengths, which place some important limitations on their practical applicability. For example, long read sequencing could enable more complete and efficient *de novo* assembly of complex genomes ( Goodwin *et al.*, 2016), and may conceivably allow for efficient haplotype phasing to characterise clinically-relevant mutations ( Goodwin *et al.*, 2016; Hussain, 2015). For RNA sequencing applications, a more reliable method for profiling the alternative isoform composition of complex transcriptomes might also be possible ( Steijger *et al.*, 2013).

The development of long read sequencing methods using ‘nanopores’ has in fact been the focus of several academic laboratories for over two decades ( Akeson *et al.*, 1999; Cherf *et al.*, 2012; Derrington *et al.*, 2010; Kasianowicz *et al.*, 1996; Meller *et al.*, 2000; Stoddart *et al.*, 2009). More recently, the technique has been further developed and commercialised by Oxford Nanopore Technologies (ONT) ( Brown & Clarke, 2016). ONT sequencing works by placing a nanopore, which in the case of the ONT platform is currently a protein pore, in a conducting electrolyte solution and applying a small potential difference across the pore. Nucleotide kmer-specific signatures of current fluctuations as a nucleic strand passes through a nanopore are then recorded to determine the sequence. A critical consideration is the size and characteristics of the sensing aperture of the pore, which determines how many nucleotides present in the pore contribute to the recorded current ( Derrington *et al.*, 2010). With smaller/more optimal sensing apertures, less nucleotides influence the characteristics of the recorded current, making distinguishing nucleotide sequences subject to much less noise. Thus using an optimally structurally configured protein pore for sequencing is a key determinant of sequencing accuracy. A second critical factor that influences sequencing accuracy, as well as throughput, is the speed and manner of DNA translocation through the pore, and in order to exert control over such parameters motor enzymes that are able to ratchet DNA into the pore at a suitable speed are employed ( Cherf *et al.*, 2012).

Thus far ONT have only commercially released their entry-level miniature ‘MinION’, currently marketed as a pocket-sized portable sequencing device, and some notable successes have been achieved that have taken advantage of its portability ( Quick *et al.*, 2015; Quick *et al.*, 2016). However, given the superiority in producing long reads, it may also be useful to consider the applicability of the MinION, and thus the ONT platform in general, for more general laboratory research. Previous benchmarking works have reported limited success in yielding combined sequence accuracy and throughput ( Ip *et al.*, 2015; Laver *et al.*, 2015), but these published studies have utilised older versions of protein pores referred to by ONT as ‘R6’ or ‘R7’. While the identity of the R6/R7 pores remain undisclosed, a low raw sequencing accuracy meant that reads needed to be sequenced in ‘2D’, where two complementary DNA strands are joined by a hairpin adapter allowing for their sequential sequencing through the nanopore. More recently, ONT have released an ‘R9 series’, which they have revealed is based on the CsgG bacterial amyloid secretion pore ( Brown & Clarke, 2016). Presumably with a more optimally configured sensing aperture, the CsgG pore is reportedly capable of higher sequencing accuracy. A mutant form of this pore, ‘R9.4’, is the current version favoured by ONT, reportedly currently yielding the highest accuracies and pore stability. Such developments in improving raw sequence accuracy could also potentially mean that 1D sequencing, and thus higher throughput, might be possible without accuracy levels falling unacceptably low. In addition, new motor enzymes that are capable of ratcheting the DNA through pores at higher speeds have also been made available, adding further potential for increased throughput. Here we describe the use of the CsgG R9.4 nanopore system, in use with a sequencing chemistry that operates a translocation speed of 250b/s, to robustly produce long sequence reads from native human genomic DNA obtained from human HAP1 cells.

## Methods

### Cell culture and genomic DNA extraction

Early passage HAP1 cells (Horizon Discovery) were grown in Iscove’s Modified Dulbecco’s Medium (Thermo Fisher Scientific) supplemented with 10% fetal bovine serum (Thermo Fisher Scientific), and maintained at a temperature of 37°C in a humidified incubator with 5% CO _2_. Cells were harvested by washing in PBS and then incubating with Trypsin-EDTA followed by further washing of detached cells in PBS. The PureLink Genomic DNA Mini Kit (Invitrogen) was used to isolate purified genomic DNA, as per the manufacturer’s instructions.

### Library preparation and sequencing

In general, we followed ONT protocols for library preparation. Wherever significant deviations were made to their recommendations, these are indicated by an asterix and are explained as a note.

500ng of genomic DNA was fragmented by shearing through a 15 gauge needle* 10 times. Damage to DNA was then repaired using the preCR repair mix (NEB)**, according to manufacturer recommendations and DNA subsequently purified using AMPureXP beads (Beckman Coulter). End-repair of DNA fragments was then performed using the Ultra II End Prep module (NEB). Ligation of ‘E7’ motor protein-complexed AMX adapter (ONT, NSK007)*** to genomic DNA ends was next carried out using the NEBNext Ultra II Ligation Module (NEB)****. Another round of AMPure XP purification was then performed before the DNA library was eluted and loaded onto a running buffer-primed flow cell for sequencing. Sequencing of the native genomic DNA was performed on a single R9.4/FLO-MIN106 flow cell on a MinION Mk1B for 30 hours and base-calling performed using the cloud-based Metrichor/EPI2ME platform. EPI2ME split reads into a ‘pass’ folder containing high quality reads and a ‘fail’ folder containing low quality reads.

*To enable generation of long fragments, we utilise DNA shearing through a 15 gauge needle, as opposed to g-tube shearing which is recommended in the ONT protocol.

**Although we performed sequencing of native DNA without PCR, we use this DNA repair step primarily for repairing of nicks that may otherwise disrupt the sequencing of long fragments.

***Although the ONT NSK007 kit was designed for 2D sequencing, we were able to adapt it for 1D sequencing by omitting the addition of the Hairpin Adapter (HPA) during the library preparation workflow.

****ONT workflows recommend the use of the Blunt/TA ligation module (NEB) for ligation of adapters. We instead use the Ultra II ligation module (NEB), which is more compatible with end-repair components performed in the previous step, and further allows the intervening wash step to be omitted.

### Computational analysis

The base-called pass/high quality and fail/low quality reads were processed separately through poretools (0.6.0; https://poretools.readthedocs.io/en/latest/) ( Loman & Quinlan, 2014) to obtain the embedded FASTQ data. The respective FASTQ data were mapped to the reference human genome (GRCh38 Primary Assembly; GenBank: GCA_000001405.15) using GraphMap (0.3.1; https://github.com/isovic/graphmap) ( Sović *et al.*, 2016) with the --sensitive parameter enabled on a High Performance Computing cluster. High quality reads were mapped in 2d:17h using 14 threads. The resulting alignments were processed using samtools (1.4; http://www.htslib.org/) ( Li *et al.*, 2009) to obtain basic mapping statistics and BAM files visualised using Tablet (1.16.09.06; https://ics.hutton.ac.uk/tablet/) ( Milne *et al.*, 2013).

**Figure 1.**
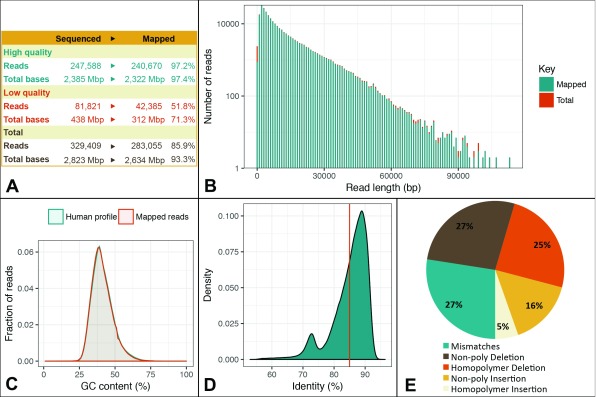
Overview of throughput and quality of mapped reads. ( **A**) Summary of the sequencing output and mapping performance. General alignment characteristics are displayed in Figure S1. ( **B**) Read length distribution of high quality reads counted in 1 Kbp bins and represented on a log _10_ scale (y-axis). Note that reads shorter than 80 bp are not considered for mapping by GraphMap by default. ( **C**) GC content distribution as output by Qualimap. ( **D**) Kernel density plot displaying the distribution of mapped read identity with the mean average (85%), indicated with a vertical line. ( **E**) Pie chart breaking down the different types of errors estimated from AlignQC; further details on the context of the errors detected is provided in Figure S2.

The high quality read alignments were processed through QualiMap (2.2.1; http://qualimap.bioinfo.cipf.es/) ( Okonechnikov *et al.*, 2016) to obtain further metrics, such as GC Distribution ( Figure 1C). We employed Quinlan’s python scripts at https://github.com/arq5x/nanopore-scripts ( Quick *et al.*, 2014) to determine identity to reference ( Figure 1D) and complementary metrics, such as alignment profiles ( Figure S1). A more detailed estimate of the error types was also obtained using AlignQC (1.2; https://www.healthcare.uiowa.edu/labs/au/AlignQC/) ( Weirather *et al.*, 2017) as summarised in Figure 1E (also see Figure S2).

Coverage data were extracted using samtools and bedtools (2.26.0; http://bedtools.readthedocs.io/en/latest/#) ( Quinlan & Hall, 2010) and plotted using R with ggplot2 and reshape2 packages ( R Core Team, 2016; Wickham, 2007; Wickham, 2009). Gene coordinates were obtained from the GENCODE Release 25 Primary GFF annotation ( https://www.gencodegenes.org/releases/25.html). To visualise the influence of coverage on consensus quality with current ONT chemistry and methodology, we performed random downsampling of the mitochondrial genome alignments (which presented the best coverage) using samtools to obtain a range of different coverages. Consensus calling was then performed using Ivan Sović’s majority consensus calling script ( https://github.com/isovic/samscripts/src/consensus.py) ( Sović *et al.*, 2016) with a minimum coverage of 1, returning the consensus differences with the reference.

## Results

### Yield and mapping characteristics

The experimental strategy employed in this study was aimed at optimising combined MinION output with regards to throughput, read-length and mapping identity from a limited amount (500ng) of non-reference (a cancer genome) starting DNA material. Though ultra-long reads (>100kb) are theoretically possible on the ONT platform, we opted for needle shearing for fragmentation of genomic DNA with the aim of yielding long sequence reads, the computational analysis tools for which have been previously optimised ( Sović *et al.*, 2016). To further improve throughput we employed a 1D sequencing strategy (see Methods); though this would inevitably lead to a loss in base-calling accuracy compared to 2D sequencing, we reasoned this would likely be compensated by improvements in raw sequencing accuracy offered by the new ONT CsgG R9.4 system employed.

From the single 30 hour MinION run of HAP1 native genomic DNA, a total of ~329,000 1D reads comprising ~2.8 billion bases were sequenced ( Figure 1A). ~247,000 were high quality reads, and of these ~97% successfully mapped to human reference genome. Thus ~240,000 mapped high quality reads consisting of ~2.3 billion bases were taken forward for analysis in this study. The read length distribution yielded a mean of ~9.6kb and an N50 of ~17kb, and the longest successfully mapped read obtained was ~113kb in length ( Figure 1B). No negative bias in terms of sequencing GC-rich sequences was apparent in our dataset ( Figure 1C). A mean mapping identity of 85% to reference was achieved from the 1D reads obtained from the native cancer genome ( Figure 1D), and most called errors were either mismatches or deletions including homopolymer-associated deletions ( Figure 1E). Indeed, ONT protein nanopores have previously generally displayed difficulties in resolving homopolymers that exceed the sensing aperture length of the pore ( Jain *et al.*, 2016), and our results further suggest that this problem persists in data generated using the current R9.4 nanopore.

**Figure 2.**
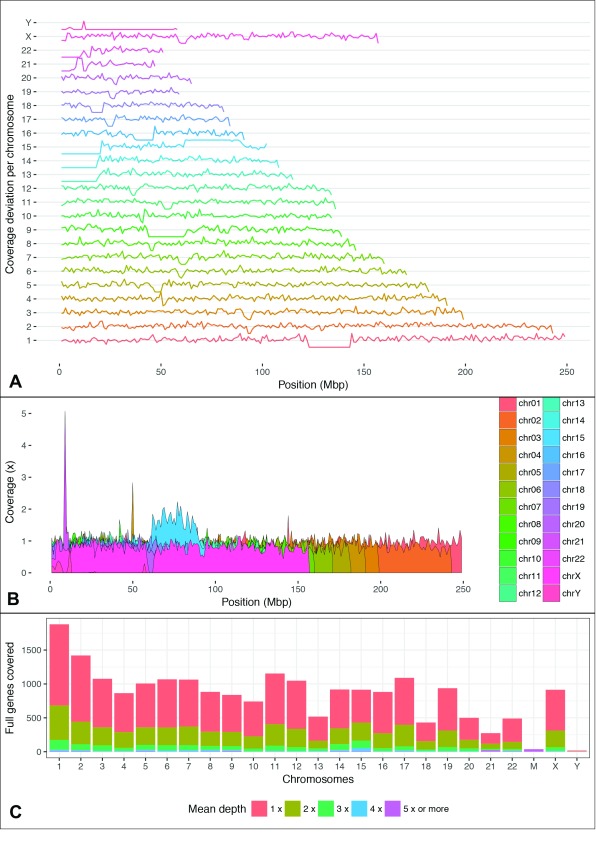
Overview of coverage achieved over the genome. ( **A**) Coverage deviation per million base pairs for each chromosome. Horizontal gridlines represent the average coverage (0.8 ×) with values ranging between a minimum and maximum range of -2 and +2 standard deviations from the mean. ( **B**) Average coverage per million base pairs, the largest increase in chromosome 15 corresponds with the remaining diploid region of HAP1 cells. ( **C**) Counts of GENCODE 25 genes with all positions covered by at least 1 base pair and varying levels of depth for each chromosome.

Barring a 30Mb diploid region spanning a portion of chromosome 15, human HAP1 cells are a fully haploid cell line ( Carette *et al.*, 2011; Essletzbichler *et al.*, 2014), and thus generally represent a particularly amenable tool for CRISPR-Cas9 mediated genome editing in potential studies of genetic function. Accordingly, coverage obtained from our dataset along the haploid genome appeared fairly uniform, except for a portion of chromosome 15 which likely corresponds to the disomic region of the genome ( Figure 2A and B). With an N50 of ~17Kb in our dataset, as expected, a significant proportion of reads covered the full length of at least a single annotated gene ( Figure 2C).

### Mitochondrial genome consensus calling

**Figure 3.**
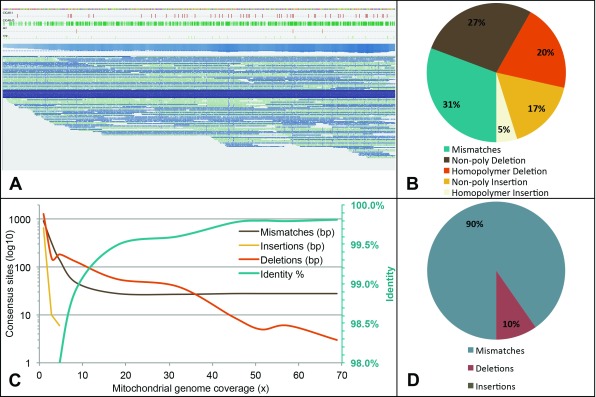
Mitochondrial genome coverage and consensus quality. ( **A**) Snapshot from Tablet, displaying the long reads mapping across the entire mitochondrial genome. Single reads covering the entire length are represented in dark blue. Several tracks also display (from the top) reference sequence, insertion sites (red), deletion sites (green), consensus deletions (orange), consensus mismatches (light green) and coverage. ( **B**) Breakdown of the different error types as estimated by AlignQC for the mitochondrial mapping only. ( **C**) Change in error counts (on a log _10_ scale) and percent identity of the final mitochondrial consensus sequence over a range of coverage levels downsampled from the full alignment. Note the percent identity scale is to the right of the graph in green. ( **D**) Breakdown of the basic error types remaining in the final full coverage consensus.

Notably, a particularly high density of reads mapped to the ~17kb mitochondrial (mt) genome, for which we achieved 68x coverage. Most of the mtDNA reads covered a significant portion of the mitochondrial genome, with a few reads indeed each covering the entire genome ( Figure 3A). The mtDNA reads mapped with 84% identity to reference, and as with our observations for the nuclear genome, most called errors were either deletions or mismatches ( Figure 3B). Since we had obtained a significant degree of coverage for the mitochondrial genome, we proceeded to inspect how errors can affect final consensus calling at increasing coverage levels, which at full coverage achieves 99.8% mean identity to reference ( Figure 3C). Our analysis revealed that ~10X coverage was required to prevent most random mismatches from being called, beyond which mismatches at specific positions remained ( Figure 3C). Insertions made up a smaller proportion of the total called errors ( Figure 3B), and required a lower coverage for complete correction ( Figure 3C). Deletions, which included homopolymer deletions, required a much greater degree of coverage, and some deletions still remained with the highest level of coverage achieved ( Figure 3C). However, following consensus calling, the majority of the total called errors remaining were in fact mismatches ( Figure 3D). Since we observed that mismatch error trend halted at ~10X coverage, it is quite likely that the remaining mismatches in fact represent genuine non-reference bases, i.e. they are a true reflection of the native DNA sequenced. Such non-reference nucleotides are likely to include unrepaired DNA damages and single nucleotide variants, and possibly also naturally modified bases.

## Discussion

Here we describe the sequencing of native DNA obtained from the near-haploid human cancer cell line HAP1. Utilising recently made available sequencing pores and chemistries, we report significant improvements in combined throughput and raw sequencing accuracy compared to that previously published using older pore-type and sequencing chemistry versions ( Ip *et al.*, 2015; Laver *et al.*, 2015). Further, in anticipation that 1D sequencing may represent the future direction taken by ONT, we modified the workflow of the early-release CsgG NSK007 kit in order to enable 1D sequencing. While we observed an 85% mapping identity of sequenced bases from 1D reads, there are a couple of relevant considerations. Firstly, we sequenced native DNA, and thus DNA modifications and unrepaired DNA damages were unlikely to be base-called correctly. Secondly, we used a cancer cell line with unknown identity to reference. 85% is therefore likely a slight underestimate of raw 1D accuracy using the sequencing chemistries employed in this study. ONT have indeed now revealed a new sequencing mode, referred to as 1D ^2^, enabling higher accuracies in the ~95% range with even further improvements anticipated in future ( https://nanoporetech.com). All such advances, including those described in this work, have been centred on the newly-employed CsgG nanopore which is thus likely to remain a stable feature of ONT protein-pore sequencing.

We also observed a robust generation of long sequence reads in our study and using our fragmentation protocol, a mean read length of ~9.6kb with an N50 of ~17kb was achieved. We opted for needle shearing as a demonstration of a quick and inexpensive method for random DNA fragmentation; however, it may be possible that even longer mappable reads can also robustly be obtained by performing library preparation without an intentional fragmentation step. Indeed, thousands of high quality reads of more than 50kb in length were mapped in our study, further suggesting that robust ‘ultra-long’ read generation should also be readily possible on the platform.

Following consensus calling, we observed 99.8% mean consensus identity in reads mapped to the mtDNA genome, for which we had obtained 68X sequencing coverage. Our analysis suggested that the majority of remaining non-reference bases in our mtDNA reads were likely genuine features of the native DNA, indicating that remaining true errors may in fact have been potentially reduced to near-zero levels. However, it should be noted that this observed high efficiency in consensus calling might well have been in part owing to the simple nature of the mtDNA genome. Homopolymer associated error-correction in more complex genomes, for example, may prove less efficient, and efforts focussed on optimising sequencing chemistries and base-calling parameters to help resolve such issues would be most welcome. Nonetheless, for applications associated with genetic variant calling, high levels of sequence throughput and coverage obtained during ONT sequencing could conceivably compensate for its currently lower raw sequencing accuracy compared to short-read platforms. Since the release of the early 250b/s prototype (now obsolete), currently a faster 450b/s translocation chemistry is available which can potentially generate up to 10–20GB of sequence from a single MinION flow cell ( https://nanoporetech.com). ONT have also begun the beta-testing phase for their ultra-high throughput benchtop sequencer, the PromethION, reportedly capable of yielding 6TB of sequence from a single 24-hour run ( Jain *et al.*, 2016). It is likely that high-throughput, long-read sequencing on the ONT platform may enable significant advances in genetics applications relevant to complex genomes to be made in the near future.

## Data availability

Sequence data used for analysis in this study is publicly archived at the European Nucleotide Archive (ENA) under accession code ERR1898537. Files contain high quality sequence data, as well as associated alignment data.

## Supplementary material

**Figure S1: Alignment profile of mapped reads.** Dotplot generated using Quinlan’s nanopore scripts ( https://github.com/arq5x/nanopore-scripts; ( Quick *et al.*, 2014)), which incorporates two factors for each read, the fraction of the read aligned and the identity (or number of matches versus alignment length + insertions). Note that a wide spread of read fractions aligned may be expected when aligning a cancer genome to reference.

**Figure S2: Detailed error contexts from AlignQC.** AlignQC output displaying if there was a bias towards mismatches/deletions/insertions in specific contexts. The error contexts seem to differ somewhat from those published in the AlignQC paper ( Weirather *et al*., 2017); such differences have likely arisen largely owing to the nature of different material sequenced, i.e. cDNA in the Weirather *et al.* study and native cancer genome DNA in ours.
