# Supplementary figures and images for: Robust long-read native DNA sequencing using the ONT CsgG Nanopore system
Source: Wellcome Open Res. 2018 Aug 30;2:23. Originally published 2017 Apr 6. [Version 3] doi: 10.12688/wellcomeopenres.11246.3 (PMC5426553; doi:10.12688/wellcomeopenres.11246.3)

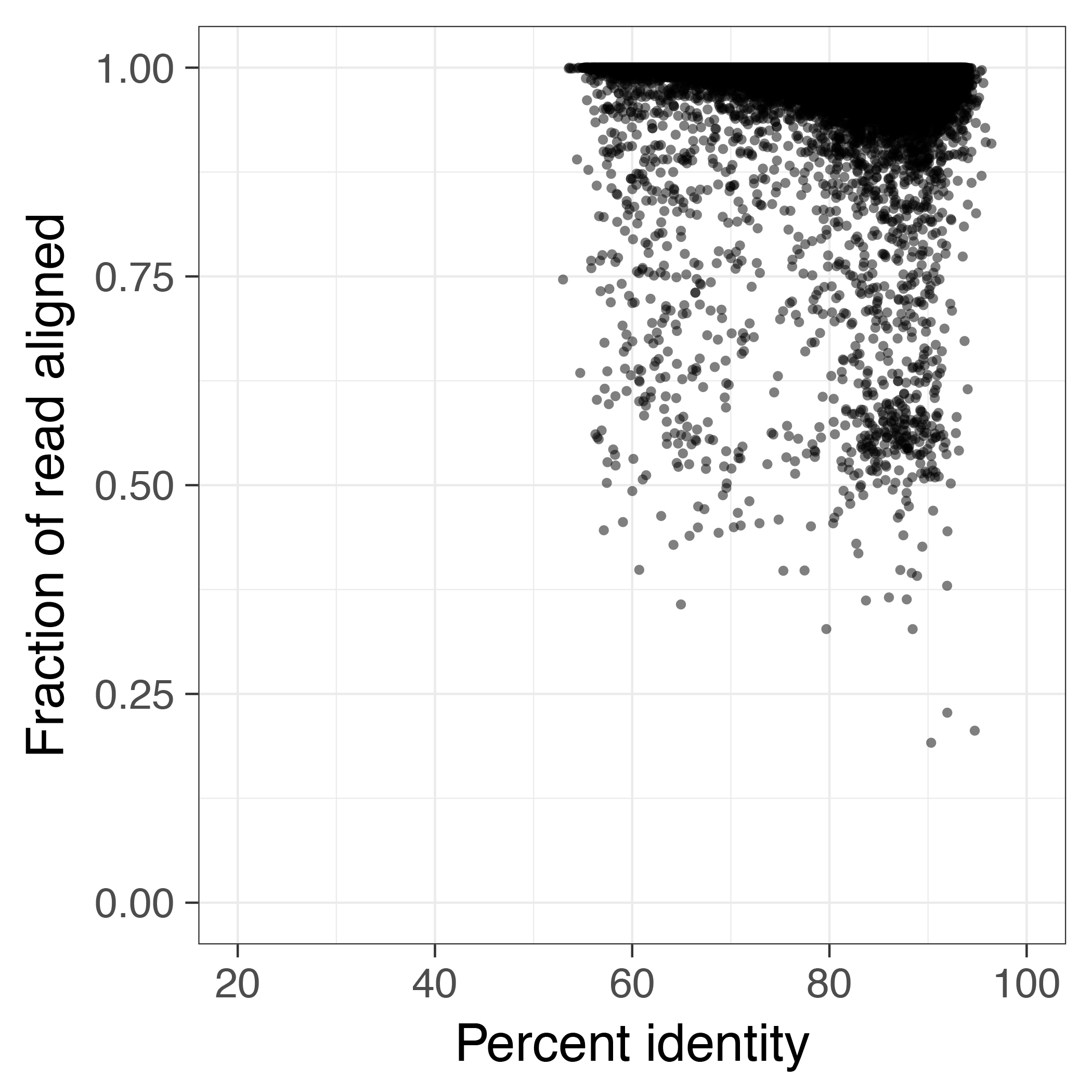

Supplement: Supplementary file 1 [file wellcomeopenres-2-16062-s0000.tgz › 0c3f1492-0620-46d7-b043-03d6f130a923.tif]

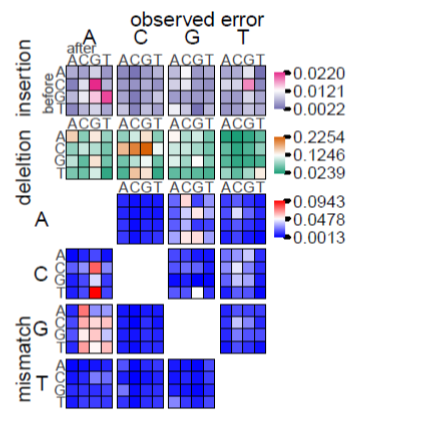

Supplement: Supplementary file 2 [file wellcomeopenres-2-16062-s0001.tgz › 315a0631-0227-48fc-ae35-e5afdbfa4bec.tif]
