# Supplementary material for: Robust long-read native DNA sequencing using the ONT CsgG Nanopore system
Source: Wellcome Open Res. 2018 Aug 30;2:23. Originally published 2017 Apr 6. [Version 3] doi: 10.12688/wellcomeopenres.11246.3 (PMC5426553; doi:10.12688/wellcomeopenres.11246.3)
Supplement: Peer review history

# Peer review: Referee response for version 1

**DOI:** 10.21956/wellcomeopenres.12133.r22292

**Published:** 2017-05-09

**Authors:** Miten Jain

**Affiliations:** 1 Department of Biomolecular Engineering, Genomics Institute, University of California, Santa Cruz, Santa Cruz, CA, USA

Carter and Hussain have presented MinION nanopore sequencing and analysis results using R9 chemistry. Their choice of HAP1 as DNA substrate is an interesting one, given its (mostly) haploid nature. The error rate measurements in this study agree with measurements reported by other MinION users for R9 chemistry. Given the rapid evolution of MinION sequencing, data from the newer R9.4 MinION chemistry (and soon to arrive R9.5) are superior than R9 data.

 However, this study will be useful to the nanopore community in form of library protocols as well as computational analysis tools.

I have read this submission. I believe that I have an appropriate level of expertise to confirm that it is of an acceptable scientific standard.

# Peer review: Referee response for version 1

**DOI:** 10.21956/wellcomeopenres.12133.r21615

**Published:** 2017-04-21

**Authors:** Mick Watson

**Affiliations:** 1 Division of Genetics and Genomics, Edinburgh Genomics, The Roslin Institute, University of Edinburgh, Edinburgh, UK

Carter and Hussain present details of the use of Oxford Nanopore's MinION, specifically R9.4 chemistry using the CsgG pore, to sequence HAP1. Though this particular sequencing kit has been surpassed by a faster kit, nevertheless the description of the sequencing serves as an adequate guide for MinION data quality and potential methods for analysing MinION data

I have read this submission. I believe that I have an appropriate level of expertise to confirm that it is of an acceptable scientific standard.
